# Single-Cell RNA-Sequencing Reveals Interactions between Endometrial Stromal Cells, Epithelial Cells, and Lymphocytes during Mouse Embryo Implantation

**DOI:** 10.3390/ijms24010213

**Published:** 2022-12-22

**Authors:** Luhan Jiang, Dandan Cao, William S. B. Yeung, Kai-Fai Lee

**Affiliations:** 1 Department of Obstetrics and Gynaecology, Li Ka Shing Faculty of Medicine, The University of Hong Kong, Pokfulam, Hong Kong SAR, China; 2 Shenzhen Key Laboratory of Fertility Regulation, The University of Hong Kong-Shenzhen Hospital, Futian District, Shenzhen 518053, China

**Keywords:** embryo implantation, endometrial stromal cells, single-cell RNA sequencing, endometrial epithelial cells, uterine NK cells, CD8^+^ T Cells

## Abstract

The decidualization of endometrial stromal cells (ESCs) is an essential process facilitating embryo implantation. However, the roles of non-decidualized and decidualized ESCs in regulating the microenvironment of a receptive endometrium remain unclear. We investigated single-cell transcriptomic changes in the uterus of a CD-1 mouse model at the post-implantation stage. The implantation and inter-implantation sites of the uteruses of pregnant mice at 4.5 and 5.5 days post-coitum were dissected for single-cell RNA sequencing. We identified eight cell types: epithelial cells, stromal cells, endothelial cells, mesothelial cells, lymphocytes, myocytes, myeloids, and pericytes. The ESC transcriptome suggests that the four ESC subtypes are involved in the extracellular remodeling during implantation. The trajectory plot of ESC subtypes indicates embryo implantation that involves a differentiation pathway from undifferentiated ESCs (ESC 1) to decidualized ESCs (DEC ESCs), with distinct signaling pathways between the ESC subtypes. Furthermore, the ligand-receptor analysis suggests that ESCs communicate with epithelial cells and immune cells through nectin and ICAM signaling. Collectively, both decidualized and non-decidualized ESCs may regulate the endometrial microenvironment for optimal endometrial receptivity and immune tolerance. This study provides insights on the molecular and cellular characteristics of mouse ESCs in modulating the epithelial and lymphocyte functions during early embryo implantation.

## 1. Introduction

The uterus contains different cell types, including epithelial cells, stromal cells, myometrial cells, and immune cells that cooperate to support embryo implantation, growth, and development [1]. During the menstrual cycle, human endometrial stromal cells (ESCs) undergo decidualization to prepare for embryo implantation. The progesterone-dependent decidualization process leads to morphological and functional changes in the ESCs [2]. In mice, decidualization is initiated by the attachment of the blastocyst to the luminal epithelium at the anti-mesometrial pole of the uterus 3.5 days post-coitum (dpc). The decidualized ESCs (DEC ESCs) then differentiate into epithelioid-like cells, forming the primary decidualization zone (PDZ) around the blastocyst from 4.5 to 5.5 dpc [3]. The PDZ acts as an avascular barrier that protects the embryo from maternal lymphocytes and other harmful agents from the maternal circulation [4]. Previous immunohistochemical studies have shown that macrophages and uterine natural killer (uNK) cells are rarely detected in the PDZ [5]. Moreover, it was demonstrated that immunoglobins cannot pass through the PDZ during early pregnancy in rats [6,7]. After the blastocyst invades the endometrium at 5.5 dpc, the epithelial cells in the implantation chamber undergo apoptosis [8]. Meanwhile, the decidualization of the ESCs continues and expands to form a secondary decidualization zone on the outer layer of the PDZ and mesometrial pole. These ESCs function to modulate the immune response, hormone production, and angiogenesis [9].

Studies have shown there are differential spatiotemporal gene expression patterns in DEC ESCs. For example, Kruppel-like factor (KLF) 5 was found to be abundantly expressed in proliferating stromal cells in the PDZ [10]. Heart- and neural crest derivative-expressed protein 2 (*Hand2*) mRNA level was increased in the anti-mesometrial (AM) pole after blastocyst implantation and under progesterone stimulation [11]. The secreted frizzled-related protein 4 (*Sfrp4*) transcript was found to be localized in the outer layer of the stromal cells as a ring connected to the myometrium [12]. Moreover, Bmp2 and Wnt4, two well-known mouse decidualization markers [3], are expressed in PDZ, beginning at 4.5 dpc and continuing to increase during pregnancy [3,13]. Noggin (Nog), a Bmp2 antagonist, is strongly expressed in the sub-epithelial stromal cells before blastocyst attachment, and rapidly decreases with the increasing Bmp2 level after implantation [14]. Scribble (Scrib), an important polarity protein, was found to regulate decidualization, as the ablation of *Scrib* in stromal cells decreased *Bmp2* and *Wnt4* mRNA expression in pregnant mice at 4.5 dpc [3].

In the endometrium, DEC ESCs communicate with other cell types to mediate embryo implantation. The communication between DEC ESCs and endometrial epithelial cells (EECs) has been reported to involve HAND2 and STAT3, as mice with ESCs lacking both *Hand2 and Stat3* develop a non-receptive endometrium [15]. During embryo implantation, the luminal epithelial cells of the endometrium lose polarity via an epithelial-to-mesenchymal transition that facilitates embryo invasion. In line with this, the deletion of Msx1 or Msx2 in mouse ESCs caused high apical-basal polarity in the epithelium, epithelial breakdown, and embryo invasion failure, leading to unsuccessful implantation [16]. 

Macrophage and uNK cells are two well-studied immune cell types in the endometrium that regulate embryo implantation. Moreover, DEC ESC-secreted TGF-β, which can maintain the differentiation of decidual NK (dNK) cells, increases the expression of CD9, CD103, and killer cell immunoglobulin-like receptors (KIR) [17]. The DEC ESCs can also secrete IL15, which then recruits uNK cells to the decidua and induces the expression of CD56 in dNK cells [17]. The CD56^+^CD16^−^NK cells are non-cytotoxic, whereas CD56^−^CD16^+^ NK cells are cytotoxic [18]. The main functions of CD56^+^CD16^−^dNK cells during early pregnancy include the promotion of tissue remodeling, angiogenesis, and trophoblast invasion in humans [19]. The dNK cells also produce IL10, which further promotes the differentiation of macrophages [20]. Macrophages can be classified as M1 and M2, which are mainly responsible for pro-inflammatory and anti-inflammatory responses, respectively. The DEC ESCs secrete chemokines to attract macrophages, and they induce macrophage polarization into M2 via CXCL4 [21]. During decidualization, the endometrial macrophages undergo M2 polarization, resulting in CD206^+^M2-like macrophages in mice during implantation [22]. The macrophages in the decidua mainly function to regulate tissue homeostasis by removing apoptotic cell debris, and they also modulate maternal immune tolerance for allogenic embryo implantation [19]. Besides macrophages, DEC ESCs can induce T regulatory cells to modulate maternal immune tolerance during early pregnancy via TGF-β signaling [23]. In addition, DEC ESCs can silence T cell-attracting chemokine expression, which limits cytotoxic CD3^+^ T cell recruitment to the decidua [24]. 

Single-cell RNA sequencing (scRNA-seq) is a powerful tool to investigate transcriptomic changes in a cell population. It has been used to analyze the mouse uterus transcriptome on postnatal day 12, which identified eight main cell types that are involved in postnatal development [25]. As stromal cell decidualization is an important biological process for embryo implantation, growth, and development, we hypothesize that both decidualized and non-decidualized ESCs, in a pregnant mouse uterus, take part in regulating the endometrial microenvironment for the optimal receptivity for embryo implantation. To investigate if both decidualized and non-decidualized ESCs regulate embryo implantation, we compared the transcriptome profiles of the implantation sites (ISs) and inter-implantation sites (I-ISs) in the uteruses of CD-1 mice at 4.5 and 5.5 dpc via scRNA-seq. We also investigated the interaction between ESCs and other cells types to identify potential signaling pathways that regulate endometrial receptivity and embryo implantation. 

## 2. Results

### 2.1. Single-Cell RNA-Sequencing of thePregnant Mouse Uterus

**Figure 1:**
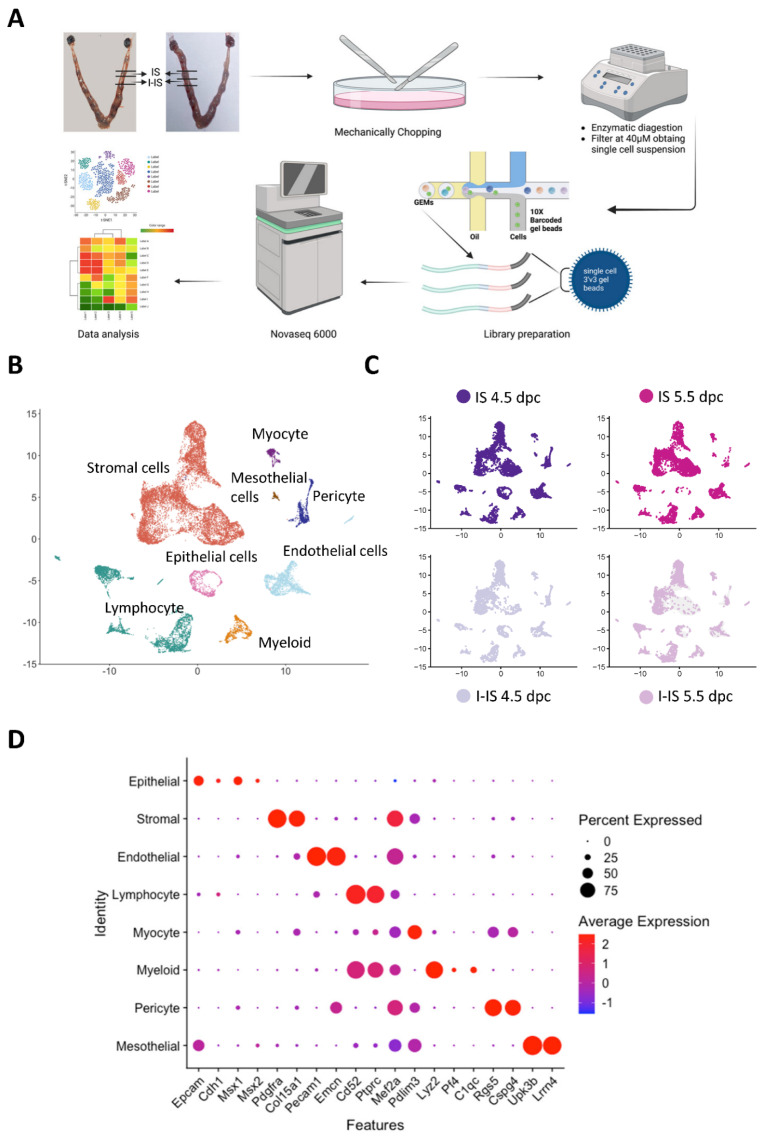
Single-cell RNA sequencing (scRNA-seq) of pregnant mice uteruses at 4.5 and 5.5 days post coitum (dpc). (**A**) Schematic diagram of the experimental design and sample preparation for scRNA-seq. The implantation sites (ISs) and inter-implantation sites (I-ISs) in the mice uteruses were collected at 4.5 and 5.5 dpc. After mechanical chopping and enzyme digestion with trypsin and collagenase, respectively, the single-cell suspensions were collected and filtered through a 40-µm cell strainer. Library preparation of the four samples was performed using a 10× Genomics Library construction kit in the Centre of PanorOmic, at the University of Hong Kong. The scRNA-seq was performed on a NovaSeq 6000 and the sequencing results were analyzed by Seurat and Monocle2 packages in R. (**B**) A UMAP plot showed eight main cell types in the pregnant mouse uterus at 4.5 and 5.5 dpc: epithelial cells, stromal cells, mesothelial cells, endothelial cells, myocytes, pericytes, lymphocytes, and myeloids. (**C**) UMAP plots showing cell distributions of IS and I-IS samples at 4.5 and 5.5 dpc. (**D**) Dot plot showing the expression of markers in different cell types. The transcripts used for annotating the eight cell types are mesothelial cell markers (*Lrrn4* and *Upk3b*), myocyte markers (*Pdlim3* and *Mef2a*), pericyte markers (*Cspg4* and *Rgs5*), myeloid markers (*C1qc*, *Pf4*, and *Lyz2*), epithelial cell markers (*Msx2*, *Msx1*, *Cdh1*, and *Epcam*), lymphocyte markers (*Ptprc* and *Cd52*), endothelial cell markers (*Emcn* and *Pecam1*), and stromal cell markers (*Col15a1*, *Dcn*, and *Pdfgra*).

To understand the transcriptional changes in different cell types in pregnant mice uteruses, we performed scRNA-seq on cell suspensions from ISs and I-ISs at 4.5 and 5.5 dpc (Figure 1A). The scRNA-seq data were generated using a 10× genome library preparation kit on a NovaSeq 6000. After quality control, a total of 18,149 cells with 200–10,000 genes per cell fulfilled the requirements for the downstream analysis (Table S1 and Figure S1). Uniform manifold approximation and projection (UMAP) analysis was performed on the top 5000 differential genes, which identified 25 cell clusters (Figure S2). The four samples (IS and I-IS at 4.5 and 5.5 dpc) were integrated into one UMAP plot via Seurat and analyzed according to the published cell type genes markers, which identified eight main cell types (Figure 1B). All eight cell types can be seen in each sample in the individual UMAP plots shown in Figure 1C. The eight cell types (and their gene markers) were mesothelial cells (*Lrrn4* and *Upk3b*), myocytes (*Pdlim3* and *Mef2a*), pericytes (*Cspg4* and *Rgs5*), myeloids (*C1qc*, *Pf4*, and *Lyz2*), epithelial cells (*Msx2*, *Msx1*, *Cdh1*, and *Epcam*), lymphocytes (*Ptprc* and *Cd52*), endothelial cells (*Emcn* and *Pecam1*), and stromal cells (*Col15a1*, *Dcn*, and *Pdfgra*) (Figure 1D).

### 2.2. Endometrial Stromal Cell Subtypes in the Pregnant Mouse Uterus

**Figure 2:**
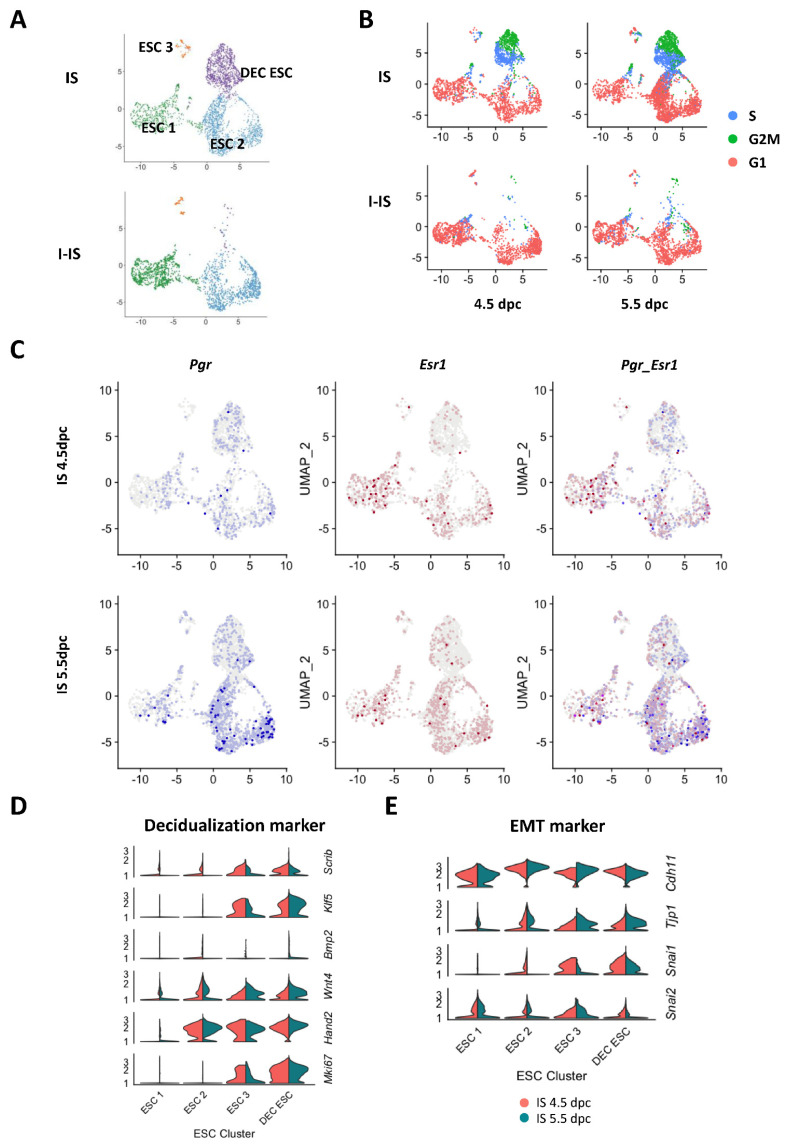
Endometrial stromal cell (ESC) sub-populations in ISs and I-ISs at 4.5 and 5.5 dpc. (**A**) ESCs were selected for UMAP plot analysis, which identified four cell subtypes (ESC 1, ESC 2, ESC 3, and DEC ESC). The DEC ESCs (purple dots) were abundant in the IS but not in the I-IS. (**B**) Cell cycle analysis showed that DEC ESC and some ESC 3 subtypes were in the G2/M (green) or S (blue) phases, indicating that they are proliferating cells. The ESC 1 and ESC 2 were mainly in the G1 (red) stage. (**C**) UMAP plots show the expression of progesterone receptor (*Pgr*) and estrogen receptor alpha (*Esr1*) on the implantation sites at both 4.5 and 5.5 dpc. The mRNA level of PGR in ESC 2 in IS at 5.5 dpc was significantly increased compared to in IS at 4.5 dpc (*p* < 0.001, *t*-test). (**D**) Violin plots show the expression of decidualization markers in the four ESC clusters. The transcription levels of the decidualization stromal cell markers *Scrib, Klf5, Bmp2, Wnt4*, and *Hand2*, and the proliferation marker *MKi67* in mouse IS on both 4.5 and 5.5 dpc. (**E**) Violin plots show the expression of epithelium-mesenchymal transition (EMT) markers *Snai1* and *Snai2,* and mesenchymal-epithelium transition (MET) markers *Tjp1* and *Cdh11* in the four ESC clusters. Statistical analysis via *t*-test. * *p* < 0.05, ** *p* < 0.01, *** *p* < 0.001.

The ESCs in the pregnant mice uteruses were re-clustered into four subtypes: ESC 1, ESC 2, ESC 3, and DEC ESC (Figure 2A). By comparing the UMAP plots between IS and I-IS, the DEC ESC cluster was found to be present only in the implantation site samples (Figure 2B). Cell cycle analysis showed that half of the DEC ESCs were at the S phase and the other half were at the G2/M phase, suggesting they were proliferating cells (Figure S3). The ESC 1 and ESC 2 clusters were at the G1 phase, whereas the ESC 3 cluster contained both proliferating and non-proliferating cells (Figure 2B). The ESC 1 and ESC 2 clusters at the implantation sites at both 4.5 and 5.5 dpc contained *Pgr-* and *Esr1*-positive cells. The expression level of *Pgr* in ESC 2 increased from 4.5 to 5.5 dpc (*p* < 0.001, Figure 2C). To further characterize the ESC subtypes, we examined the expression of decidualization markers *Bmp2, Wnt4, Scrib, Klf5*, and *Hand2*. The Violin plot analysis showed that the ESC 3 and DEC ESC clusters had higher levels of *Scrib, Klf5, Wnt4, and Hand2* transcripts (Figure 2D). Specifically, the levels of *Scrib and Hand2* were increased in DEC ESCs at 4.5 dpc (*p* < 0.001), whereas *Wnt4* and *Klf5* were highly expressed in DEC ESCs at 5.5 dpc (*p* < 0.001) (Figure 2D). Notably, ESC 2 expressed both *Hand2* and *Wnt4* at 4.5 and 5.5 dpc, but *Scrib* was highly expressed only at 4.5 dpc (Figure 2D). On the other hand, the ESC 1 cluster demonstrated fewer decidualization markers (Figure 2D). The proliferation of ESCs was determined by the presence of Mki67, with higher levels of *Mki67* in ESC 3 and DEC ESCs at 4.5 dpc compared to 5.5 dpc (*p* < 0.01), which was concordant with the cell cycle analysis (Figure 2D). The expression of the mesenchymal-epithelial transition (MET) markers is shown in Figure 2E. Both *Cdh11* and *Tjp1* are epithelial markers, whereas *Snai1* and *Snai2* are genes involved in EMT. All of the ESC subtypes highly expressed *Cdh11* at both 4.5 and 5.5 dpc, whereas only the ESC2, ESC 3, and DEC ESC subtypes highly expressed *Tjp1*. The levels of *Snai1* in the ESC2, ESC 3, and DEC ESC subtypes decreased from 4.5 to 5.5 dpc, indicating a loss of the mesenchymal characteristics during decidualization (*p* < 0.001). On the other hand, *Snai2* was detected in all of the ESCs subtypes, although the levels decreased from 4.5 to 5.5 dpc in the DEC ESCs (*p* < 0.001).

### 2.3. Differentiation Trajectories and Functions of ESCs during Early Embryo Implantation

**Figure 3:**
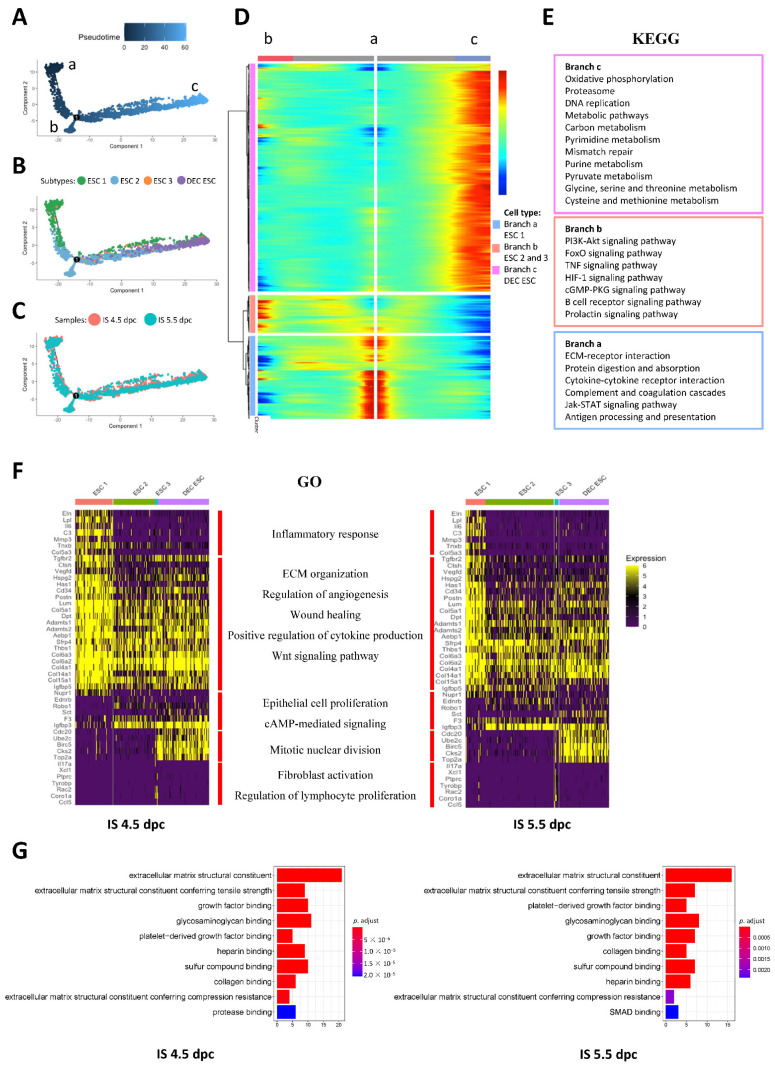
Functional analysis of ESC subtypes during early mouse embryo implantation. The trajectory plots of the ESCs colored using (**A**) pseudotime, (**B**) ESC clusters (ESC 1-3 and DES ESC), and (**C**) cell origin (IS 4.5 and IS 5.5 dpc). In the pseudotime plot, the three differentiation branches (branches a, b, and c) correspond to root, sub-branch, and termination of differentiation, respectively. (**D**) The branched expression analysis modeling (BEAM) of the two branches in pregnant mouse ESC trajectory. Top 2000 differentially expressed genes (*p* < 0.01) along the pseudotime were divided into three gene clusters based on their expression levels in the three differentiation branches and visualized in the BEAM plot. (**E**) KEGG analysis of the three gene clusters in the BEAM plot for the enrichment of the signaling pathways to determine ESC differentiation during embryo implantation in mice. (**F**) The selected differentially expressed gene (*p* < 0.05 and average log2(FC) > 1) relating to each ESC subtype (ESC 1-3 and DEC ESC) function and their gene ontology (GO) terms. ESC 1 was mainly responsible for inflammatory responses, ESC 2 was mainly involved in ECM remodeling and angiogenesis regulation, ESC 3 was mainly involved in fibroblast activation and the regulation of lymphocyte proliferation, and the DEC ESCs were mainly responsible for cAMP signaling and mitotic nuclear division. (**G**) The GO molecular function (MF) analysis of differentially expressed genes of non-decidualized ESCs in mouse uterus during implantation at 4.5 and 5.5 dpc (*p*.adjust < 0.05, the *p* value was adjusted using the Benjamini–Hochberg method).

To understand the differentiation trajectories of ESCs, we performed a pseudotime trajectory analysis by allocating the cells onto a pseudotime path based on the transcriptome similarities using the Monocle2 package in R (Figure 3A–C). Most of the ESC 1 subtypes resided at the root (branch a) of the trajectory, whereas the DEC ESC subtype was the most differentiated (branch c) and had the highest pseudotime score (Figure 3B). Both the ESC 2 and ESC 3 subtypes were in the middle of the trajectory, although a sub-branch (branch b) was formed by ESC 2. The top 2000 differentially expressed genes (DEGs) were selected for branched expression analysis modeling (BEAM), which grouped the highly expressed genes located in each branch. 

The gene list from BEAM was then subjected to Kyoto Encyclopedia of Genes and Genomes (KEGG) analysis. At the root of the trajectory (branch a), the non-decidualized ESCs were enriched in genes related to the extracellular matrix (ECM) remodeling and immune response, which are involved in ECM-receptor interactions and antigen processing and presentation. The DEC ESCs (branch c) were enriched in genes involved in cell proliferation and DNA replication. The ESC 2 subtype involved in branch b was enriched in genes of classic decidualization regulation and function, such as the PI3K-Akt signaling and FoxO signaling pathways (Figure 3E).

Gene ontology (GO) analysis was conducted on the DEGs (*p* < 0.001 and expressed in >30% of cells) in each ESC subtype. All ESCs were enriched in genes related to ECM remodeling, angiogenesis, cytokine production, and Wnt signaling at both 4.5 and 5.5 dpc (Figure 3F). The ESC 1 subtype was specifically enriched in genes related to the inflammatory response, whereas the ESC 2, ESC 3, and DEC ESC subtypes were enriched in genes related to the regulation of epithelial cell proliferation and cAMP signaling. The ESC 3 group showed a specific transcriptome feature involving fibroblast activation and the regulation of lymphocyte proliferation (Figure 3F).

The BEAM analysis indicated that non-decidualized ESCs were actively involved in embryo implantation. We next investigated the molecular functions of DEGs in ESC 1 and ESC 2 at the IS (*p* < 0.001, genes expressed by >30% of the cells within the ESC subtype and >2 fold-change between subtypes). At 4.5 and 5.5 dpc, the non-decidualized ESCs were enriched in genes related to ECM remodeling and growth factor functions. Notably, non-decidualized ESCs at 5.5 dpc were enriched in genes involved in SMAD-dependent pathways (Figure 3G). The gene ontology analysis of the biological processes of DEGs in non-decidualized ESCs showed similar results (Figure S4). Overall, the results suggest that both the decidualized and non-decidualized ESCs play important roles in mediating embryo implantation.

### 2.4. Cell-Cell Interactions between ESC Subtypes in the Pregnant Mouse Uterus

**Figure 4:**
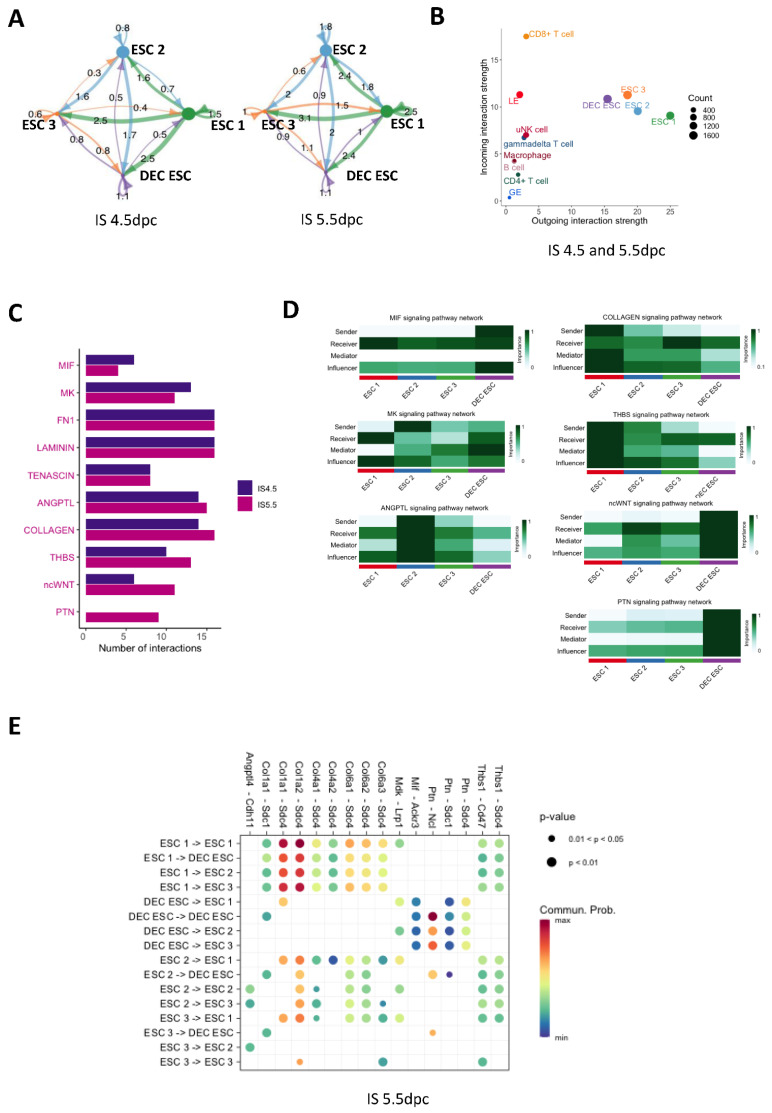
Ligand–receptor interactions between ESC subtypes in mouse implantation sites. (**A**) The shell plots show cell-cell interactions in the four ESC subtypes (ESC 1-3 and DEC ESC) in the IS at 4.5 and 5.5 dpc. (**B**) The scatter plot shows the outgoing and incoming interaction strengths of the ESCs and immune cells in the IS at 4.5 and 5.5 dpc. The circle size represents the number of interaction counts. (**C**) The number of ligand–receptor interactions of selected interactions in the IS at both 4.5 and 5.5 dpc. (**D**) The heat map shows the relative importance of each ESC subtype based on the computed four network centrality measures of seven differential expressed signaling networks between 4.5 and 5.5 dpc, including thrombospondin (THBS), collagen, midkine (MK), angiopoietin-like proteins (ANGPTL), non-canonical WNT (ncWNT), pleiotrophin (PTN), and migration inhibitory factor (MIF) pathways. (**E**) All significant ligand–receptor pairs contribute to the signaling in the four ESC clusters. The dot color and size represent the calculated communication probability and *p* values (one-sided permutation test).

To understand the interactions between the four ESC subtypes during embryo implantation, we used CellChat to predict the ligand-receptor interactions. The ligand receptor pair ratios between each ESC subtype are shown as shell plots in Figure 4A. The pair ratios indicate that the intensities of the cell interactions at 4.5 and 5.5 dpc were similar (Figure 4A). Moreover, the strong cell–cell interactions of non-decidualized ESCs suggest that they are important in embryo implantation. By comparing the incoming and outgoing signal strength between ESCs, EECs, and immune cells, we can see that the ESCs contributed the most outgoing signals, whereas CD8^+^ T cells received the most incoming signals (Figure 4B). The differentially regulated signaling pathways between 4.5 and 5.5 dpc were the migration inhibitory factor (MIF), midkine (MK), collagen, thrombospondin (THBS), non-canonical WNT (ncWNT), and pleiotrophin (PTN) pathways (Figure 4C and Figure S5). The network centrality analyses of these pathways are shown in Figure 4D. For the non-decidualized ESCs, ESC 1 was the dominant sender, mediator, and influencer of collagen and THBS signaling to itself and to other ESC subtypes (Figure 4D). The ESC 2 group showed differentiation, but it was not decidualized based on the trajectory plot (Figure 3A). MK and ANGPTL signaling were the dominant pathways controlling the non-decidualized ESC subtypes (Figure 4D). The DEC ESCs were found to control ESC 2 via ncWNT signaling, and they were the main sender and influencer of MIF signaling in the non-decidualized ESC subtypes. The PTN pathway was the major signaling pathway controlling cell interactions in the DEC ESCs, but only at 5.5 dpc (Figure 4C–D). The detailed ligand-receptor molecules in the above signaling pathways are shown in the bubble plot in Figure 4E. Interestingly, the Col1a1/Col1a2-Sdc4 signals between ESC 1 and other ESC subtypes, as well as Ptn-Ncl signals in the DEC ESCs, showed the most intense interactions (Figure 4E).

### 2.5. Interactions between ESCs and EECs at the Implantation Site at 4.5 dpc

**Figure 5:**
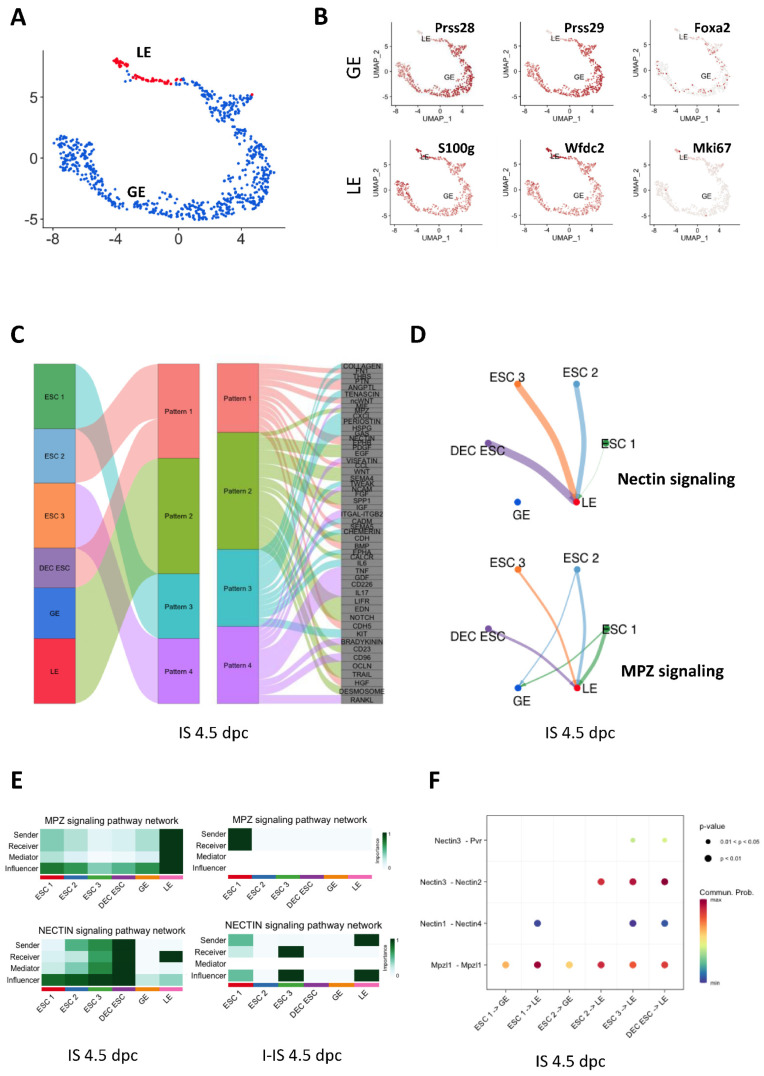
The endometrial epithelial cells (EECs) in mice uteruses and their interactions with the ESCs during early embryo implantation at 4.5 dpc. (**A**) The UMAP plot shows luminal epithelium (LE, red) and glandular epithelium (GE, blue) in the mouse endometrium during embryo implantation in the combined 4.5 and 5.5 dpc samples. (**B**) The expression patterns of the GE markers (*Prss28, Prss29*, and *Foxa2*), LE markers (*S100g* and *Wfdc2*), and proliferation marker Mki67 were projected onto UMAP plots. (**C**) The outgoing communication patterns of the ESCs (ESC 1-3 and DEC ESC) and EECs (GE and LE) showing correspondence between the interaction patterns and each cell subtype and the signaling pathways involved. (**D**) The shell plot shows the interaction of nectin and MPZ signaling between ESCs (ESC1-3 and DEC ESC) and EECs (GE and LE). (**E**) The heat map shows the relative importance of each ESC and EEC subtype based on the computed four network centrality measures of seven differential expressed signaling networks between ISs and I-ISs at 4.5 dpc. (**F**) All of the significant ligand–receptor pairs that contribute to the signaling from each ESC subtype to EEC subtype are shown in a dot plot. The dot color and size represent the calculated communication probability and *p* values (*p* values are computed from the one-sided permutation test).

The EECs were re-clustered in a UMAP plot (Figure 5A). The interactions between the EEC and the ESC clusters were analyzed through CellChat using the *Prss28*, *Prss29*, and *Foxa2* transcripts as glandular epithelium (GE) markers, and S100g and Wfdc2 as luminal epithelium (LE) markers (Figure 5A,B). Due to the apoptosis of LE in the endometrium at 5.5 dpc, we analyzed only the 4.5 dpc sample. The outgoing signaling patterns of each ESC and EEC (GE and LE) subtype are shown in the river plot. Notably, ESC 2 and the DEC ESCs shared the same outgoing signaling patterns, indicating their similar roles in mediating endometrial homeostasis during implantation (Figure 5C). By comparing the signaling patterns between the IS and I-IS at 4.5 dpc, we identified two differential signaling pathways, namely, the myeline protein zero (MPZ) and nectin pathways. The strength of the signaling from the ESCs to the EECs is shown in the shell plot in Figure 5D. The detailed heatmap plot further showed that LE was the major sender, receiver, mediator, and influencer of the MPZ signaling in the IS, whereas ESC 1 was the major sender and receiver of the signaling in the I-IS. The DEC ESCs played a main role in the nectin signaling communication to the LE (Figure 5E). Further analyzing the two pathways showed that Mpz-like 1 (Mpzl1)-Mpzl1 and nectin3-nectin2 were the most intense ligand receptor pairs in the ESC–EEC interactions (Figure 5F).

### 2.6. The Transcriptomic Landscape of Lymphocytes during Embryo Implantation

**Figure 6:**
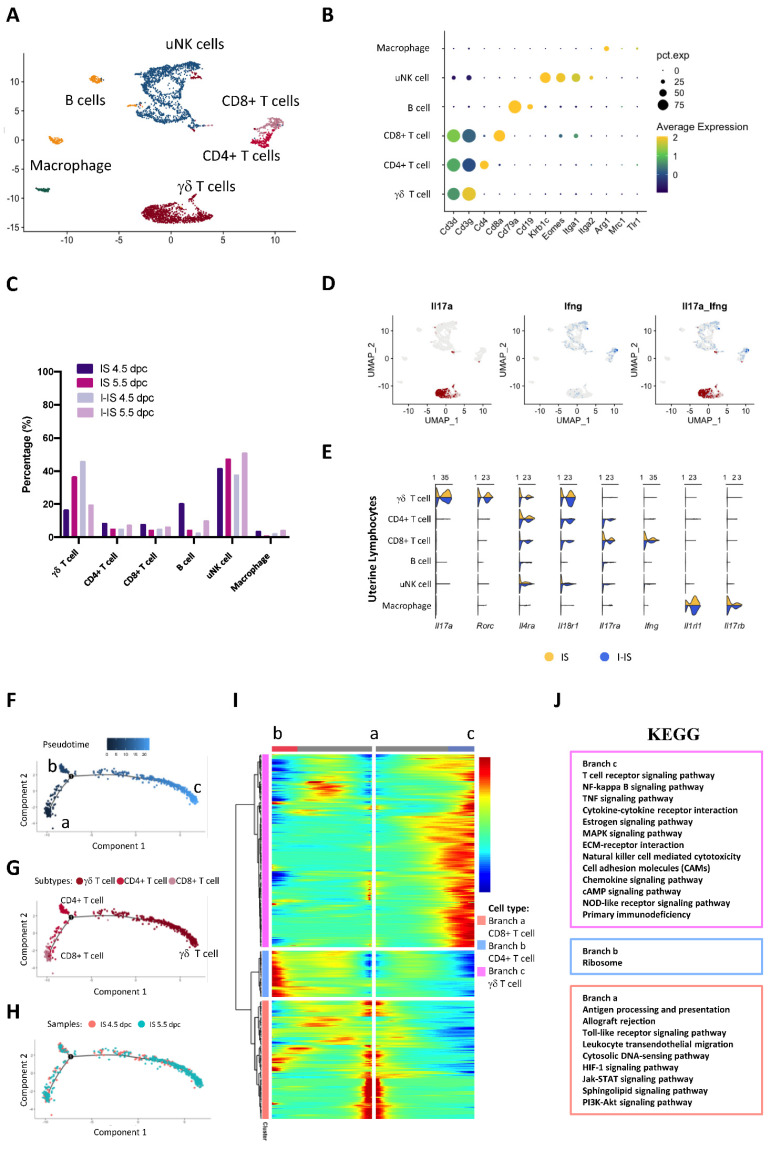
Lymphocyte populations and their functions in pregnant mice uteruses during embryo implantation. (**A**) The different lymphocyte populations in pregnant mice uteruses in the UMAP plot shows distinctive clusters of T cells, B cells, macrophage, and uterine natural killer (uNK) cells. (**B**) The dot plot shows the expression levels of cell type-specific markers of T cells (*Cd3d,* and *Cd3g*), Cd4^+^ T cells (*Cd4*), CD8^+^ T and B cells (*Cd79a*), uNK cells (*Klrb1c*, *Eomes, Itga1,* and *Itga2*), and macrophages (*Arg1*, *Mrc1*, and *Tlr1*). (**C**) The UMAP plot shows the distribution of the lymphocytes from the ISs and I-ISs at 4.5 and 5.5 dpc. (**D**) The UMAP plot shows the transcription levels of the cytotoxicity marker *Ifng* and type 2 immune response marker *Il17a* in pregnant mice uteruses. (**E**) The Violin plot shows the transcription level of the cytotoxic markers and their receptors (*Il17a, Rorc, Il4ra, Il18r1, Il17ra, Ifng, Il1rl1,* and *Il17rb*) in lymphocytes between IS and I-IS (combined 4.5 and 5.5 dpc samples). (**F**-H) The trajectory plots of T cell subtypes colored using (**F**) pseudotime, (**G**) T cell subtypes, and (**H**) cell origin. (**I**) BEAM analysis of the two branches of the pregnancy trajectory. The top 700 genes differentially expressed (*p* < 0.01) along the pseudotime were divided into three gene clusters based on their expression level in the three differentiation branches shown in the BEAM plot. (**J**) KEGG analysis of the three gene clusters in the BEAM plot to confirm that the enriched signaling pathways showed T cell differentiation during mouse embryo implantation.

The four main types of lymphocytes were identified in the early IS, including T cells, B cells, uNK cells, and macrophages. The UMAP plot showed that these cells were clustered into distinctive groups (Figure 6A). Their specific marker expressions are shown in the dot plot in Figure 6B (references are listed in Table S2). The distribution of lymphocytes from each sample is shown in the UMAP plot in Figure 6C. The T cells and uNK cells contributed the highest proportion of lymphocytes in the early pregnant mice uteruses. The levels of type II immunity cytokine receptors, including *Il4ra*, *Il18r*, *Il17rb*, and *Il10r* mRNA, are presented in Figure 6E. The γδT cells highly expressed *Il17a* and *Rorc,* but not *Ifng* (Figure 6D–E). The IL-17^+^ γδT cells expressed both pro- and anti-inflammatory immune cytokine receptor transcripts, including *Il18r1*, *Il17ra*, *Il17rb*, *Il4ra*, and *Il1rl1* (Figure 6E). Both T cells and uNK cells expressed *Il18rl* in the in I-IS at 4.5 and 5.5 dpc. The receptor of IL4, *Il4ra,* was expressed in the γδT and CD4^+^ T cells in both the IS and I-IS, and in CD8^+^ T and B cells in the I-IS (Figure 6E). The receptor of IL33, *Il1rl1*, was expressed in macrophages. The IL17 receptors, *Il17ra* and *Il17rb,* were differentially expressed in the early pregnant mice uteruses. The mRNA of *Il17ra* was mainly detected in the CD8^+^T, B, and uNK cells (Figure 6E), the transcript of *Il17rb* was highly expressed only in macrophages (Figure 6D), and *Il17a* was expressed only in γδT cells (Figure 6E). The majority of uNK cells at the implantation sites were activated (Figure S6); they were mostly Eomes^+^CD49a^+^DX5^+/−^, and a small group were Eomes^+^CD49a^−^DX5^+^ (Figure S7). The macrophages in early pregnant mice uteruses expressed the surface markers *Arg1* and *Tlr1*. Based on the subtype markers of the macrophage, the macrophages in the pregnant mice uteruses showed a mixed M1/M2 profile expressing both the M1 markers, such as Hif1a, Irf3, Stat1, Nkfb1; and M2 markers, such as Maf, Klf1, and Stat (Figure S8). The B cells in the mouse embryo implantation sites expressed *Cd79*, *Cd19*, *Cd24a*, and *Cd38* markers (Figure 6B and Figure S9).

The results of the trajectory analysis of T cells are presented by pseudotime, cell subtypes, and cell origin in Figure 6F–H. Cytotoxic CD8^+^ T cells were located at the root of the trajectory, whereas γδT cells were located at the end (Figure 6G). To identify the DEGs during T cell differentiation, a BEAM analysis of 707 DEGs (*p* < 0.01) was conducted using Monocle2 (Figure 6I). The KEGG analysis revealed that the active signaling pathways of T cells were located in each of the different branches (Figure 6J). The γδT cells were actively involved in the NF-κB, hormone response, ECM-receptor pathways, and cytokine or chemokine interactions (Figure 6J). The CD4^+^ αβT cells were located at branch b, which played a role in embryo antigen recognition and presenting, and modulated the immune-suppressive response via PI3K-AKT and sphingolipid signaling (Figure 6J).

### 2.7. Cell Interactions between ESCs and Lymphocytes

**Figure 7:**
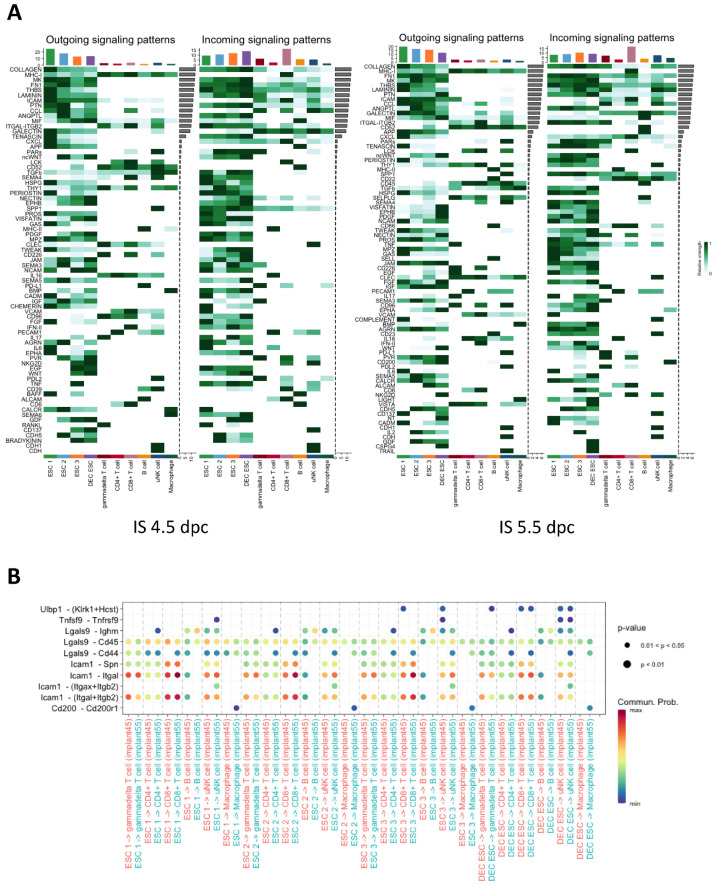
The ligand–receptor interaction between the ESCs and uterine lymphocytes during early mouse embryo implantation. (**A**) The outgoing and incoming signal patterns of ESC and lymphocyte subtypes in the implantation sites at both 4.5 and 5.5 dpc. (**B**) Comparison of the significant ligand–receptor pairs between the ESCs and lymphocytes in the implantation sites between 4.5 and 5.5 dpc. Dot color reflects the communication probabilities, and dot size represents the computed *p* values. The *p* values were computed from the one-sided permutation test. Empty squares show that the communication probability is zero.

The cell–cell interactions between the ESC subtypes and immune cells were analyzed by using CellChat. The heat map shows the potential signaling pathways actively involved in the communication between the ESCs and immune cells (Figure 7A). The bar chart at the top of the heat map shows the intensity of cell communication for each cell type (Figure 7A). The ESCs exhibited most of the outgoing signals and CD8^+^ T cells received most of the incoming signals at 4.5 and 5.5 dpc (Figure 7A). The uNK cells, B cells, and macrophages were under the regulation of the ESCs during early embryo implantation (Figure 7A). Interestingly, ESCs regulated the CD8^+^ T cells mainly through intracellular adhesion molecule 1 (ICAM), chemokine C-X-C motif ligand (CXCL), and activated leukocyte cell adhesion molecule (ALCAM) (Figure 7A), whereas ESCs regulated uNK cells through NKG2D and CD137 (Figure 7A). Specifically, the ESC 3 and DEC ESC subtypes engaged in the ligand–receptor interactions with uNK cells through CD137 in IS at 4.5 dpc, whereas ESC 1 was the main cell type involved in this interaction in IS at 5.5 dpc (Figure 7A). Similar to CD137 interactions in IS at 4.5 dpc, there were also strong interactions between uNK cells, ESC 3, and DEC ESCs through NKG2D signaling, although only the DEC ESCs communicated with the uNK cells through NKG2D at 5.5 dpc. The ESCs expressed CD200, whereas its receptor was expressed exclusively in the macrophages. We constructed a bubble plot to obtain insights into the molecules involved in the differential interactions between the ESCs and lymphocytes, which revealed the involvement of ICAM, CXCL, ALCAM, NKG2D, CD137, and CD200 (Figure 7B). The high communication probability of ICAM-Itgal and ICAM-(Itgal+Itgab2) indicates that ESCs may modulate the functions of CD8^+^T and uNK cells during embryo implantation (Figure 7B).

## 3. Discussion

In this study, we characterized the transcription landscape of implantation and inter-implantation sites in mouse uteruses at the single-cell resolution during early pregnancy. In mice, 4.5 dpc is the time at which decidualization starts, and 5.5 dpc is the time when the PDZ is complete [3]. Although embryos enter the uterine cavity at 3.5 dpc and attach to the endometrium at 4.0 dpc [3], it is possible that floating embryos can induce the decidualization of the uterus at 3.5 dpc. However, the effects may not be detectable, and no ISs or I-ISs are formed that can be used for transcriptomic analysis. Although the uterus at 3.5 dpc could be used as a control for our transcriptomic study, the differences in the hormonal profiles will add another variable into the analysis. Therefore, we harvested and compared the ISs and I-ISs from the mouse uteruses at 4.5 dpc and 5.5 dpc in this study. To our best knowledge, this is the first study examining the roles of ESCs in the molecular regulation of tissue homeostasis in pregnant mouse uteruses from 4.5 to 5.5 dpc. The results of the study extend our understanding of the transcriptomic landscape of tissue differentiation and remodeling controlled by stromal cells and their interactions with lymphocytes during embryo implantation.

Based on the expressions of different cellular markers [25], we identified eight main cell types: epithelial cells, stromal cells, endothelial cells, mesothelial cells, lymphocytes, myocytes, myeloids, and pericytes. We found that ESCs showed transcriptional and functional heterogeneity during embryo implantation. The heterogeneity of the ESCs has also been confirmed in human endometrium in the proliferative phase [26]. Based on the transcriptomic differences, we identified four main clusters of ESCs in the implantation sites, but only three clusters in the inter-implantation sites during early pregnancy. As ESC decidualization only occurs when the mouse blastocyte attaches to the endometrial epithelium, we expected decidualized ESCs only at the implantation sites, as the other three subclusters were non-decidualized ESCs [27]. It was reported that *Wnt4* and *Hand2* are expressed in the ESCs in the PDZ of mice on day 5 of pregnancy [3]. Consistent with the previously reported in situ hybridization staining results [3,13], we found that the transcription levels of the decidualization markers *Klf5*, *Wnt4*, *Bmp2*, and *Hand2* were increased from 4.5 to 5.5 dpc. Interestingly, DEC ESCs and ESC 2 abundantly expressed *Wnt4* and *Hand2* transcripts, indicating that some non-decidualized ESCs may have important functions at the implantation site. The cell cycle analysis showed that the DEC ESCs were in the S or G2/M phase of the endocycle. In rodents, the formation of the primary decidual zone (PDZ) starts at 4.5 dpc and completes at 5.5 dpc, whereas the secondary decidualized zone (SDZ), comprising both differentiating and proliferating ESCs, starts to form at 5.5 dpc [28]. The ESCs in the PDZ highly express transcripts and proteins in the cell cycle, including cyclin D3, Cdk4, Cdk6, and p21 [28]. The cooperation between Cdk6, cyclin D3, and p21 induces ESC cell cycle arrest at the G2/M phase and triggers ESC endocycle leading to polyploidization [28]. Furthermore, cyclin D3/p21/cdk6 increases the expression of cyclin E in the SDZ, which triggers the progression of ESCs from the G to S phase of the endocycle [28]. Therefore, DEC ESCs in the S phase are likely to be from the SDZ, whereas those in the G2/M phase are from the PDZ.

The trajectory analysis of ESCs at the implantation sites at 4.5 and 5.5 dpc indicated the ESCs had three differentiation states, namely trajectory branches a, b, and c. The KEGG analysis of the DEGs in each trajectory branch showed differences in signaling during ESC differentiation. In branch c, DEC ESCs showed highly activated pathways involved in DNA replication and anti-oxidative stress, indicating that the DEC ESCs are highly replicative and that they protect the embryo and maternal tissue from stress induced by embryo implantation. Moreover, transcript levels of *Tjp1* and *Cdh11,* which encode the tight junction protein, increased from 4.5 to 5.5 dpc, indicating that the DEC ESCs are important for establishing a physical barrier to protect the endometrium from invasion by the embryo. In branch a, non-decidualized ESCs were located at the root of the trajectory, and were enriched in genes related to ECM, protein metabolism, cytokine, Jak-STAT, and antigen pathways. These results suggest that the non-decidualized ESCs are involved in tissue remodeling and in regulating the maternal immune response. In branch b, ESCs were shown to be enriched in genes related to PI3K-Akt, FoxO, TNF, HIF-1, cGMP-PKG, B cell receptor, and prolactin signaling pathways, which are classical pathways mediating ESC functions during decidualization and embryo implantation. Surprisingly, GO analysis of the exclusive markers expressed by non-decidualized ESCs suggest they are functionally active during embryo implantation. This suggests that the decidualized ESCs play roles in the immune response, ECM remodeling, and epithelial proliferation during implantation [2]. However, our dataset showed that both non-decidualized ESC 1 and ESC 2 may also participate in these processes in pregnant mice. A recent scRNA-seq study on the proliferative human endometrium suggested that non-decidualized ESCs are involved in ECM remodeling and homeostasis after menstruation for tissue repair [26].

In early pregnancy, ESCs play a central role in mediating the endometrial receptivity for implantation, while EECs undergo apoptosis, as shown by the lower *Mki67* mRNA levels at 5.5 dpc [27]. Here, we showed that ESCs were the major source of outgoing signals, while EECs and lymphocytes received the majority of the signals at 4.5 dpc. The river plot of the outgoing signals of ESCs and EECs during early pregnancy showed that different ESC subtypes secreted different ligands that are speculated to regulate EECs in mediating embryo attachment onto the luminal epithelium. It has also been suggested that epithelial-stromal communication is hormone-dependent [29]. For example, PGR signaling is involved in initiating epithelial-stromal crosstalk via inducing the Indian hedgehog homolog (IHH), which leads to the upregulated orphan nuclear receptor Chicken Ovalbumin Upstream Promoter Transcription Factor 2 (COUP-TFII) and its downstream transcription factor, heart- and neural crest derivative-expressed protein 2 (HAND2) [29]. A previous in vitro study reported that human ESCs in the proliferative phase inhibited EEC proliferation, while inducing EEC differentiation [30]. Here, we found that the ESCs may dominate the ESC–EEC interaction even in the initial stage of embryo implantation.

We compared the ligand–receptor interactions between IS and I-IS at 4.5 dpc, which identified MPZ and nectin as being differentially regulated signaling pathways. As the most abundant myelin protein in the peripheral nervous system (PNS), MPZ acts as a major cell adhesion molecule in the myelin sheaths of Schwann cells and oligodendrocytes [31]. In line with this, peripheral myelin protein-22 (PMP22), another myelin protein in the PNS has been identified in the human endometrium during both the proliferative and secretory phases [32]. It induces the expression of α6 integrin accompanied by increased binding to the ECM laminin [32]. In this study, the ligand–receptor pair in MPZ signaling within the luminal EECs was mediated by the transmembrane protein Mpzl. In cancer, Mpzl induces ovarian, colorectal, and breast tumor cell proliferation, migration, and invasion [33]. During embryo attachment, the interaction of Mpzl disassembles the adhesive complex between the luminal EECs to facilitate trophectoderm penetration [34]. Nectins are a family of cell adhesion molecules that regulate Ca^2+^-independent cellular adhesion [35]. They are functionally correlated with classic cell adhesion molecules, such as cadherins, integrins, and growth factor receptors [35]. Nectin-2 can directly recognize N-cadherin, which is highly expressed in ESCs and is important for the formation of adherens junctions [35]. Human blastocysts were shown to secrete miR-661, which significantly reduced the expression of nectin-1 in human EECs, suggesting that nectin signaling plays a vital role in mediating embryo implantation [36]. In this study, nectin signaling was highly activated between the decidualized ESCs and luminal EECs at the implantation site. Hence, nectin signaling in ESCs could be a novel target for regulating embryo implantation in mice, although further investigations are needed to understand the function of nectin in ESC–EEC communication.

We next studied the importance of ESCs in regulating the immune response in the endometrium. The immune regulation during early embryo implantation involves both pro-inflammatory and anti-inflammatory responses, as demonstrated by the presence of both types of cytokine in lymphocytes (Figure 6D,E and Figure S8). The T cells expressed *Tnf*, *Il17,* and *Tgfb1* indicating a balanced immune response during embryo implantation. Based on the T cell receptors, T cells can be grouped into conventional αβT cells and non-conventional γδT cells [37]. The αβT cells, including the CD8^+^/CD3^+^ and the CD4^+^/CD3^+^ T cells, play a key role in the adaptive immune response and recognize major histocompatibility complex (MHC) Class I and Class II protein antigens [38]. On the contrary, γδT cells, including CD8^−^/CD4^−^/CD3^+^ T cells, are abundantly located in the mucosal site, and they function in innate immune responses that are not MHC-restricted [38,39]. As they mostly reside on the epithelial surface, γδT cells contribute only 0.5–10% of the total T cell population in blood and lymphoid tissues [40]. It has been reported that γδT cells accumulate in the maternal–fetal interface [41], which was supported by our current study, which found that γδT was the major T cell at the maternal–fetal interface. There are two major types of γδT cells, namely Tγδ1 and Tγδ17 [42]. The Tγδ1 cells produce IFNγ, which is involved in type 1 immunity, whereas the Tγδ17 cells produce IL-17, which is involved in type 2 immunity [43]. Moreover, Tγδ17 cells are responsible for extracellular bacterial and fungal clearance, whereas Tγδ1 cells have roles in intracellular pathogen clearance and anti-tumor response [42]. During early pregnancy, γδT cells in the endometrium have a type 2 immunity phenotype [44]. In the current study, we found that γδT cells had increased levels of the *Il17α* and *Rorc* transcripts, which support the type 2 immunity phenotype in early pregnancy. The pro-inflammatory cytokine IL17 has a pivotal role in mediating the immune response during embryo implantation [45]. More specifically, IL17 functions to recruit immune cells, and synergistically interacts with other pro-inflammatory cytokines [46]. In a murine model, high levels of IL17 positively regulated trophoblast invasion [37]. We found higher levels of *Il17* in γδT cells in the implantation sites of CD-1 mice, which is consistent with the previous finding that γδT cells produce IL17 during gestation [47]. We showed that IL17 receptor beta (Il17rb) was expressed in macrophages, which is the receptor of IL25 and mediates type 2 immunity. Moreover, macrophage-mediated tissue repair was activated by type 2 cytokines [48]. Although mRNA levels of type 2 immune cytokines were not highly abundant in mouse embryo implantation sites, the transcripts of their receptors (e.g., Il4r, Il10r, Il17ra, Il17rb, and Il18r1) were detectable in the mouse uterus during pregnancy (Figure 6E).

Macrophages are a critical component of the host immune response [49]. Macrophages make up approximately 20–30% of all decidual leukocytes in the human maternal–fetus interface [50]. Macrophages can be classified into M1 and M2 macrophages. The M1 macrophages are pro-inflammatory and anti-microbial, whereas the M2 macrophages are anti-inflammatory and are involved in tissue repair [49]. It has been shown that CD200–CD200R interactions can induce the anti-inflammatory reprogramming of macrophages during embryo implantation [51]. Based on the mRNA levels of macrophage markers, it was speculated that macrophages in the pregnant mouse endometrium would have mixed M1/M2 profiles [52], which was supported by our findings (Figure S8A). We tested the expression of the distinct surface markers of four types of M2, namely M2a, b, c, and d, each with phenotypes and functions (Figure S8B) [53]. Macrophages in the mouse embryo implantation sites highly express the M2c marker arginase I (Arg1) [53], which can be induced by PGE2, Th2 cytokines, and cAMP, alone or synergistically [54]. In mammals, arginine metabolism is an important cellular process that determines M1/M2 polarization [54]. We speculate that the decidualization of ESCs would induce M2c polarization via CD200-CD200R to facilitate tissue remodeling during early implantation in mice.

The uNK cells represent the predominant type of lymphocyte in mouse uteruses. As a marker of NK cells, NK1.1 (*Klrb1c*) is expressed in most common mouse strains [55]. We found that the cell activation marker B220 (*Ptprc*) was highly expressed in NK1.1^+^ uNK cells in the uterus of early pregnant mouse (Figure S6). Conventional NK (cNK) cells belong to innate lymphocyte cells that distinctively express the transcription factor Eomesodermin (Eomes) [56]. In mice, the uNK cell can be divided into three subgroups based on their cell surface markers: Eomes^+^CD49a^+^CD49b^+^, Eomes^+^CD49a^−^CD49b^+^, and Eomes^+^CD49a^+^CD49b^−^ [57]. The uterine tissue-resident NK (trNK) cells are Eomes^+^CD49a^+^DX5^+/−^, with higher transcription levels of angiogenic factors, whereas cNK cells are Eomes^+^CD49^−^DX5^+^ and have higher *Ifng* transcript levels [58,59,60]. Notably, IFN-γ is an important factor for vascular remodeling during murine pregnancy [61]. In mouse early pregnancy, Eomes^+^Cd49a^+^ trNK cells are predominant in the endometrium compared with lower levels of cNK cells [62]. However, we were unable to group the uNK cells in our scRNA-seq dataset into the three subtypes based on the transcript levels of the cell surface markers (Figure S6). The transcript of *Itga1* encoding CD49 was abundantly expressed compared with the expression of *Itga2* encoding DX5 in murine uNK cells, which is consistent with previous findings (Figure S7).

The contribution of B cells to maternal immunotolerance in early pregnancy has been understudied compared to T cells and uNK cells. We showed that B cells in murine peri-implantation sites highly expressed *Cd79a*, *Cd24a*, *Cd19*, and *Cd38* transcripts (Figure 6B and Figure S9) [63,64], with the latter two markers being involved in B cell development [64,65]. The co-expression of CD24 and CD38 in humans indicates an IL10-producing regulatory B (Breg) cell phenotype. Breg cells have immunosuppressive functions via the targeting of other immune cells such as pro-inflammatory monocytes and T cells [64]. Maternal B cells are the main source of anti-inflammatory IL-10, which is essential for fetus development in the uterus [66,67]. In human early pregnancy, decidual B cells can also produce IL-10 to support the maternal immune response [68]. Thus, the strong immunosuppressive capacity of Breg cells may support a suitable endometrial microenvironment for embryo implantation [69,70]. In rodents, Breg cells can be classified into IL-10^+^ B cells, CD5^+^CD1d^+^ B cells, CD80^+^CD86^+^ B cells, CD80^+^CD86^+^CD27^+^IL^-^10^+^ B cells, IL-35^+^ B cells, and PIBF1^+^ choriodecidual B cells [69]. However, we found the CD-1 female mice highly expressed *Cd24a* and *Cd19* but not *Cd80* and *Cd86* mRNA at the embryo implantation site at 5.5 dpc. This could be due to the small number of B cells identified in the sample and the differential expression patterns between the mRNA and protein levels of Breg cell surface markers.

The cell–cell interaction analysis suggested that ESCs can also mediate the immune response. Most of the outgoing signals came from ESCs, with CD8^+^ T cells and uNK cells being the major receivers of the incoming signals. By comparing outgoing and incoming signals between each cell type, we identified ICAM, galectin, NKG2D, CD137, and CD200 as the differentially expressed signaling pathways from 4.5 to 5.5 dpc, suggesting that ESCs can suppress the immune response and strengthen maternal immune tolerance. The glycoprotein ICAM-1 is known to recruit leukocytes to the inflammation site [71] to regulate wound healing and tissue hemostasis in response to inflammation [71]. An in vitro co-culture study demonstrated that ICAM-1 can regulate receptivity and trophoblast invasion under the control of gonadotrophin [72]. In CD8^+^ T cells, ICAM-1 can inhibit the expression of IFNγ and granzyme B, which are markers of cell cytotoxicity [73]. Galectin 9 (Gal9) is a member of the animal lectin family with the ability to crosslink with glycoproteins [74]. It has an anti-inflammatory role via the induction of CD8^+^ T cell apoptosis by binding to programmed cell death protein 1 (PD-1) and T cell immunoglobulin and mucin domain-containing protein 3 (TIM-3) [74]. However, the mechanisms and effects of fibroblast-T cell crosstalk via galectin signaling under physiological and pathological conditions have not been reported. Our data suggest that ESCs have a crucial role in upregulating the maternal immune tolerance via the suppression of CD^+^8 T cell toxicity during embryo implantation. We found that NKG2D, a C-type lectin-like cytotoxicity receptor, was expressed in γδT, CD8^+^ T, and NK cells, which is consistent with previous findings [75]. The ligands of NKG2D such as UL16-binding proteins (ULBPs) can stimulate the cytotoxicity of NK cells [76]. Both ULBP1 and 2 are upregulated in the senescent human fibroblast IMR-90 [77]. Moreover, uNK cells can activate angiogenesis during implantation via IL15 secreted by decidualized ESCs [78]. The interaction of Ulbp1 and NKG2D was mainly observed between ESC 3/DEC ESCs and uNK cells/CD8^+^ T cells. Therefore, communication between ESCs and lymphocytes is important to establish a favorable maternal immune microenvironment for embryo implantation.

This study had some limitations that need to be addressed in future research. First, only transcriptomic data are shown in the manuscript. This study aimed to provide a clearer picture of the transcriptomic regulation of different cell types in mouse early pregnancy, although further validation is still needed. In particular, most of the lymphocytes were characterized by surface markers and not by the transcript level. Moreover, the transcriptomic analysis of lymphocytes at the implantation site may not be as precise as the protein level. Second, only two female mice were used at 4.5 dpc. To obtain higher-quality sequencing data, we pooled all of the IS and I-IS samples to increase the cell number for sequencing. Larger sample sizes will be needed to validate the results, as the small number of mice may lead to variations in the sequencing results. Third, only two time points were investigated during embryo implantation. In future studies, more time points during the embryo implantation process should be examined to obtain a more comprehensive transcriptomic map of the mouse uterus in early pregnancy. Finally, cell–cell interactions were based on an in-silico analysis without experimental validation. An in vitro model for studying cell–cell interaction needs to be established.

In conclusion, this study provides insights on the global transcriptomic landscape of the mouse uterus during early pregnancy. The dataset identified the important roles of decidualized and non-decidualized ESCs on mediating endometrial receptivity and immune response in embryo implantation. To our best knowledge, this is the first study to report the role of non-decidualized ESCs on embryo implantation. The lymphocytes in the mouse uterus showed immune suppressive phenotypes during implantation. By analyzing the cell–cell interactions between ESCs and lymphocytes, we speculate that ESCs mediate maternal immune tolerance via ICAM and gelatin signaling to CD8^+^ T cells as well as NKG2D to uNK cells to inhibit their cytotoxicity. We found that the main macrophages in the mouse uteruses might be mixed M1/M2 profiles. We also identified several molecules that might serve as potential targets for further understanding the processes in the endometrium during embryo implantation and trophoblast invasion. Understanding the molecular mechanism could facilitate the development of new methods or diagnostic markers to enhance the implantation rate to improve infertility. Our datasets can also be used as a reference database for further scRNA-seq study in gene-edited or drug-treated mouse models. Furthermore, by comparing transcriptomic features with other stromal cell databases, we can better understand the role of stromal cell differentiation in tissue regeneration and remodeling.

## 4. Materials and Methods

### 4.1. Sample Collection

The study protocol was approved by the Committee on the Use of Live Animals in Teaching and Research, The University of Hong Kong (CULATR No.: 5435-20). Adult female and male ICR CD-1 mice were housed under a 12 h/12 h light/dark cycle with free access to food and water. Female mice at 6–8 weeks old were mated with fertile males. The presence of a vaginal plug was counted as 0.5 days post coitum (dpc). The embryo implantation sites were visualized using Chicago Blue B dye injection. The uteruses of two mice were collected at 4.5 dpc and samples from three mice were collected at 5.5 dpc. The whole uterus was segmented into implantation sites (ISs) and inter-implantation sites (I-ISs).

### 4.2. Single-Cell Dissociation of the Mouse Uterus

The uteri from two mice at 4.5 dpc and from three mice at 5.5 dpc were dissected, collected, and pooled into respective groups. In general, there are approximately 8–10 implantation and inter-implantation sites per mouse. The ISs and I-ISs from 4.5 or 5.5 dpc were dissected via mechanical chopping, followed by digestion in 500 µL of 0.05% trypsin and 75 IU DNase I (D5025, Sigma-Aldrich, St. Louis, MO, USA) in 50 µL RPMI1640 medium (R0883, Sigma-Aldrich) on a thermomixer at 37 °C with shaking at 1000 rpm for 30 min. The second digestion was performed in 2 mg/mL collagenase V (C9263, Sigma-Aldrich) and 75 IU DNase I at 37 °C for 10 min. The cell suspension was passed through a 40-µm cell strainer (352235, Corning, Corning, NY, USA) to remove undigested tissue. Dead cells were removed using a dead cell removal kit (MACS, 130-090-101, Miltenyi Biotec Inc., Bergisch Gladbach, Germany). Cell viability and density were measured using trypan blue staining and by counting the number of cells using a hemocytometer. The single-cell suspension (>1000 cells/µL, cell viability > 90%) in PBS with 0.1% BSA was used for single-cell RNA sequencing (scRNA-seq).

### 4.3. Single-Cell RNA-Seq Library Preparation and Sequencing

The scRNA-seq library preparation and sequencing of pregnant mouse uterus samples was performed at the Centre of PanorOmic Science, at the University of Hong Kong. A total of 22,750 cells in each sample were input into the Chromium Single Cell B Chip (#1000073, 10× Genomics), with the aim being to recover 10,000 cells for sequencing. Single cells were encapsulated using the Gel Bead in emulsion using the 10× Chromium Controller. Reverse transcription was performed on the Gel Bead in emulsion [26] for cDNA synthesis. The cDNA clean-up and amplification were performed using the Chromium Single Cell 30 Library and Gel Bead Kit v3 (#1000075, 10× Genomics). The libraries were sequenced on a NovaSeq 6000 (Illumina, San Diego, CA, USA) using a 151-bp paired-end mode at a depth of approximately 500 million reads per sample.

### 4.4. Single-Cell RNA-Seq Data Analysis

The fastq files were aligned to the mouse reference genome GRCm38 using the CellRanger pipeline v3.0.1 (10× Genomics, Pleasanton, CA, USA). The aggregated cell-gene count matrices of the four samples were processed using Seurat v3.1.5 in R [79]. Low-quality cells were excluded for cells with fewer than 200 unique genes, greater than 20% mitochondrial counts, and a gene expression novelty score (log10GenesPerUMI) of lower than 0.75. Furthermore, only genes expressed in more than 10 cells were kept for the downstream analysis. After quality control, the filtered matrix was normalized and scaled using the NormalizeData and ScaleData functions in Seurat, respectively. The top 5000 genes were used in the principal component analysis (PCA). JackStraw in Seurat was performed to determine the optimal number of PCA components. Single cells were clustered using the graph-based algorithm in PCA space and further visualized using the Uniformed Manifold Approximation and Projection (UMAP) dimension reduction technique. The assignment of each cell cluster was based on the published markers of known cell types. The ESCs, EECs, and lymphocytes were further re-clustered to generate new UMAP plots. To identify the differentially expressed genes in each of the ESC subclusters, the FindAllMarkers function was applied with a minimum threshold of 30% of cells in the cluster expressing the gene and a minimum log2FC threshold of 1.

### 4.5. Pseudotime and Branched Expression Analysis Modeling (BEAM)

Pseudotime and BEAM were performed on ESCs and T cells using Monocle2 (version: 2.14.0) in R [80]. The count data and metadata from Seurat were imported into Monocle2 for constructing the CellDataSet object. Feature genes were selected by using the differentialGeneTest function. The trajectory plot of ESCs in the IS was constructed using 4876 genes with *p* < 2.33 × 10^−10^. Similarly, the trajectory plot of T cells in the IS was constructed using 1120 genes with *p* < 0.01. The dimension reduction was first conducted using the DDRTree algorithm, followed by the orderCells function to construct the trajectory. The trajectory was visualized using the plot_cell_trajectory function. To identify the genes related to cell fate during pseudotime, differentialGeneTest was used with the parameter fullModelFormulaStr = “~sm.ns(Pseudotime)” [81]. The expression level of selected feature genes in either ESCs or T cells in IS was plotted on a bidirectional heat map using the plot_genes_branched_heatmap function.

### 4.6. Gene Ontology Analysis

Gene ontology (GO) analysis was performed using clusterProfiler (version: 3.14.3) in R as previously described [82]. The GO terms were selected according to the Molecular Function category in the Bioconductor annotation package org.Mm.eg.db [83]. The hypergeometric test in enrichGO was used for enrichment analysis with adjustment via Benjamini–Hochberg corrections to control the false discovery rate. The significance threshold for identifying enriched GO terms was adjusted *p* < 0.05.

### 4.7. Kyoto Encyclopedia of Genes and Genomes (KEGG) Pathway Enrichment Analysis

The KEGG pathway enrichment analysis was conducted using the DAVID online tool [84]. The significance threshold for the KEGG analysis was FDR < 0.05.

### 4.8. Ligand-Receptor Interaction Analysis

The cell–cell interaction analysis based on the ligand–receptor interactions between ESCs, EECs, and lymphocytes was conducted using CellChat v1.4.0 [85]. CellChat was used to derive a communication probability value for each ligand–receptor pair based on the average expressions of a ligand in one cell group and receptor in another cell group according to the law of mass action. The statistical significance of the communication probability values of each ligand–receptor interaction was assessed using a permutation test. The identification of the dominant senders, receivers, mediators, and influencers in the intercellular communication networks was performed by using the netAnalysis_computeCentrality function with default settings and then visualized in a heat map. The identification of major signals for specific cell groups and global communication patterns was conducted using non-negative matrix factorization for pattern recognition and then visualized in a river plot.

The selection criteria of the signaling pathways for significant ligand–receptor interactions were based on the difference in the information flow between the ISs and I-ISs at 4.5 dpc. The signaling pathways with increased information flow in the IS at 4.5 dpc were chosen for further analysis. Information flow was the total communication probability of all of the cell pairs in a given signaling pathway [85]. The communication probability was calculated by the law of mass action. CellChat was used to project the gene expression profile onto a protein–protein network, which had been previously validated experimentally [85]. We then calculated the ligand–receptor expression levels in the two cell types (i.e., ligand in cell type A and receptor in cell type B). There are several conditions for the ligand–receptor pairs: there are multiple co-stimulatory or co-inhibitory interactions, and there are multiple extracellular agonists or antagonists. The communication probabilities among all of the pairs of cell groups across all of the ligand–receptor pairs were calculated using a three-dimensional array considering all of the conditions mentioned above [85].

### 4.9. Statistical Analysis

The transcript level of the target gene between samples was compared via a *t*-test in R. *p* < 0.05 was considered to be statistically significant.

## Data Availability

All data are available via the corresponding author.

## Supplementary Materials

The following supporting information can be downloaded at: https://www.mdpi.com/article/10.3390/ijms24010213/s1.
