# Supplementary material for: Single-Cell RNA-Sequencing Reveals Interactions between Endometrial Stromal Cells, Epithelial Cells, and Lymphocytes during Mouse Embryo Implantation
Source: Int J Mol Sci. 2022 Dec 22;24(1):213. doi: 10.3390/ijms24010213 (PMC9820401; doi:10.3390/ijms24010213)
Supplement: Supplementary file 1 [file ijms-24-00213-s001.zip › Figures_supplementary.pptx]

## Slide 1
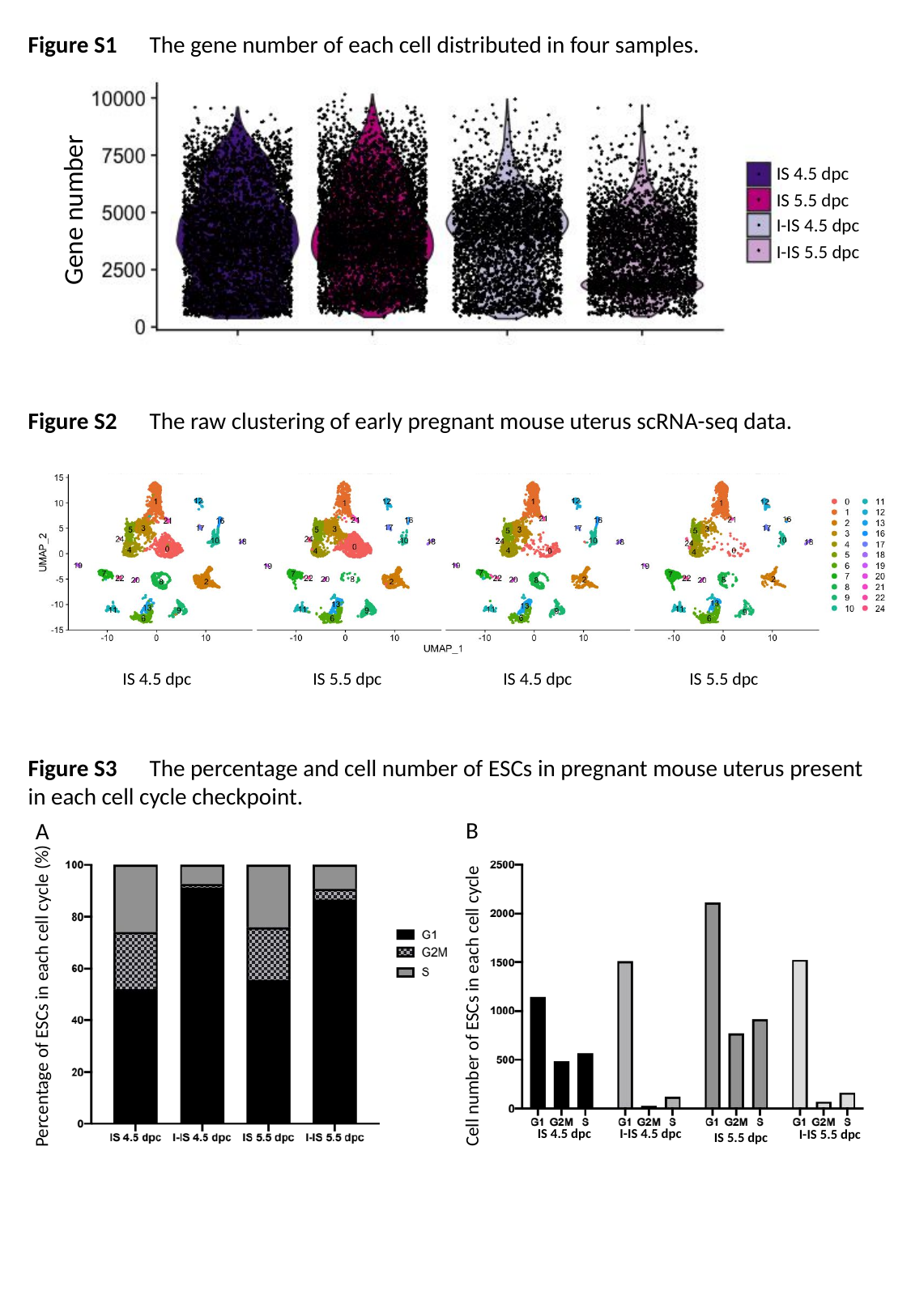

Figure S1	The gene number of each cell distributed in four samples.
IS 4.5 dpc
IS 5.5 dpc
Gene number
I-IS 4.5 dpc
I-IS 5.5 dpc
Figure S2	The raw clustering of early pregnant mouse uterus scRNA-seq data.
IS 4.5 dpc
IS 5.5 dpc
IS 4.5 dpc
IS 5.5 dpc
Figure S3	The percentage and cell number of ESCs in pregnant mouse uterus present in each cell cycle checkpoint.
B
A
Cell number of ESCs in each cell cycle
Percentage of ESCs in each cell cycle (%)
IS 4.5 dpc
I-IS 4.5 dpc
I-IS 5.5 dpc
IS 5.5 dpc

## Slide 2
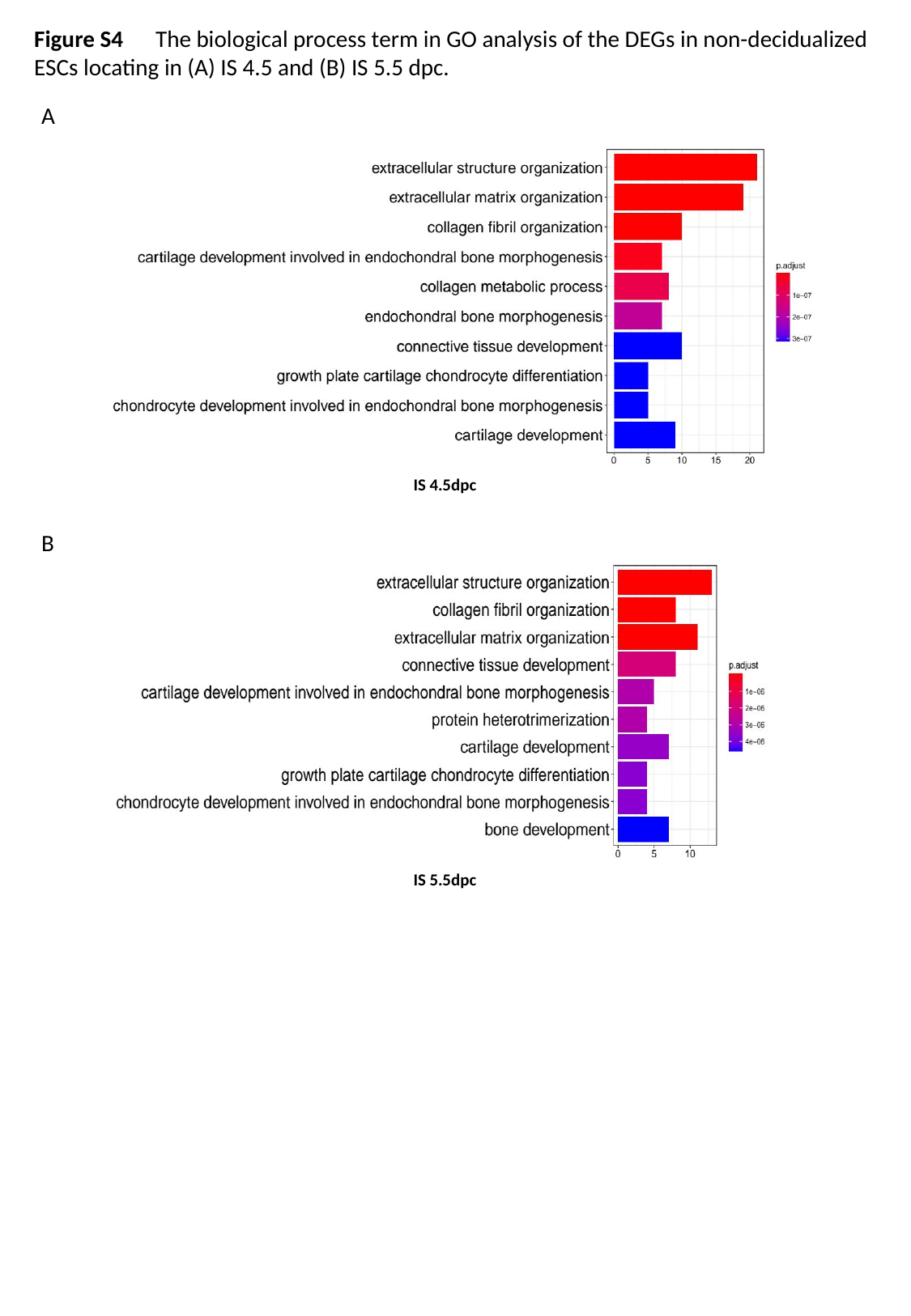

Figure S4	The biological process term in GO analysis of the DEGs in non-decidualized ESCs locating in (A) IS 4.5 and (B) IS 5.5 dpc.
A
IS 4.5dpc
B
IS 5.5dpc

## Slide 3
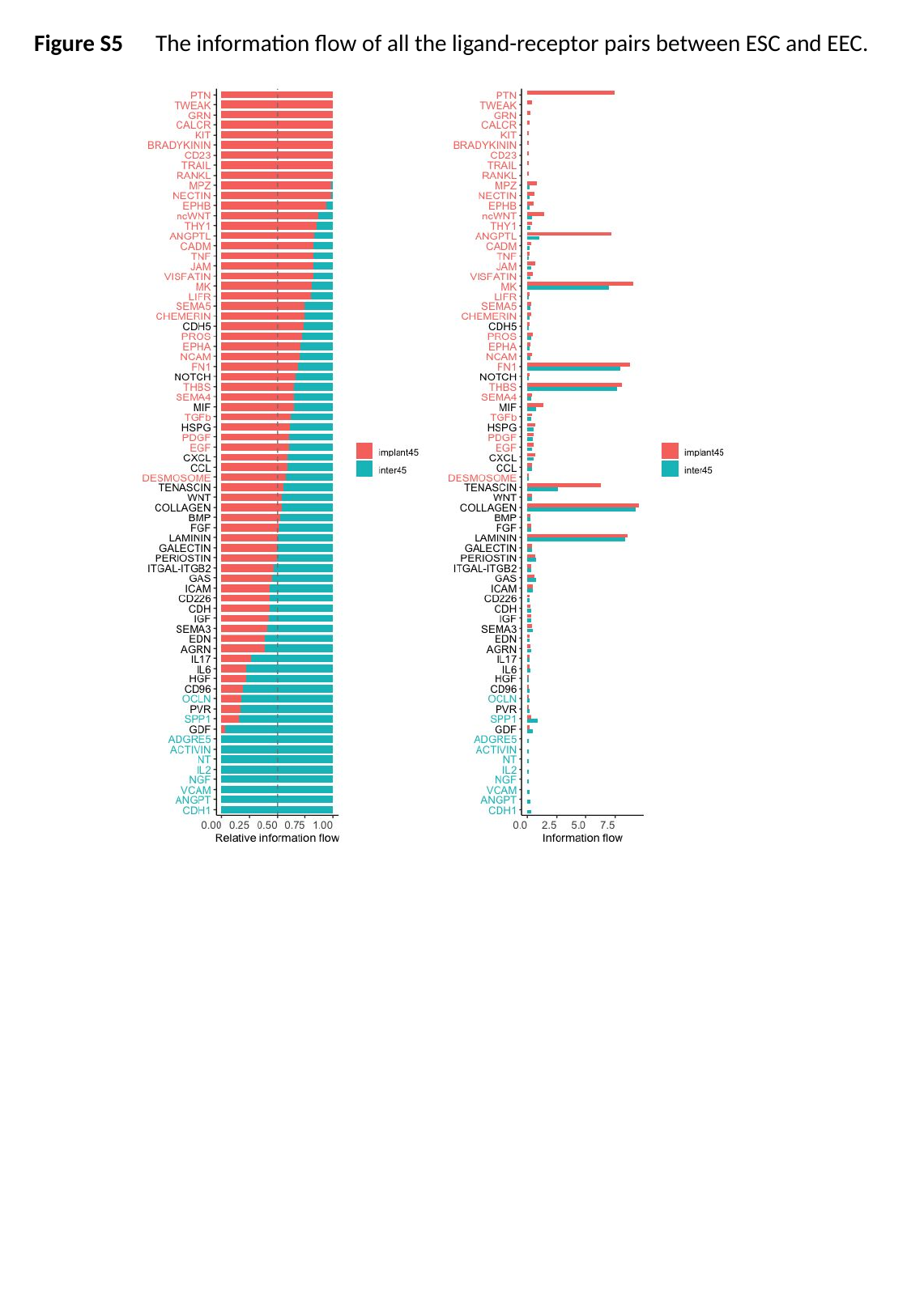

Figure S5	The information flow of all the ligand-receptor pairs between ESC and EEC.

## Slide 4
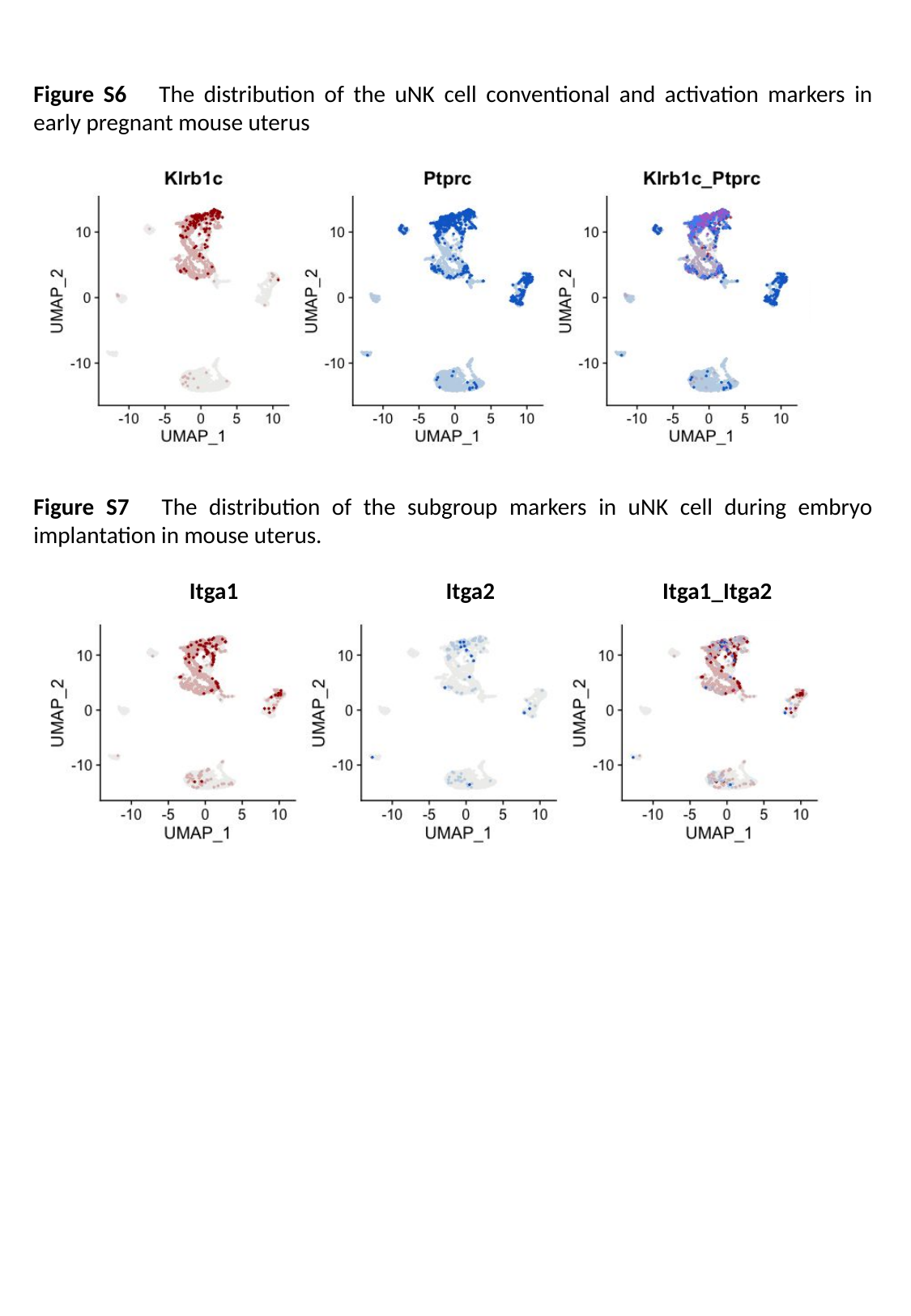

Figure S6	The distribution of the uNK cell conventional and activation markers in early pregnant mouse uterus
Figure S7	The distribution of the subgroup markers in uNK cell during embryo implantation in mouse uterus.
Itga1_Itga2
Itga2
Itga1

## Slide 5
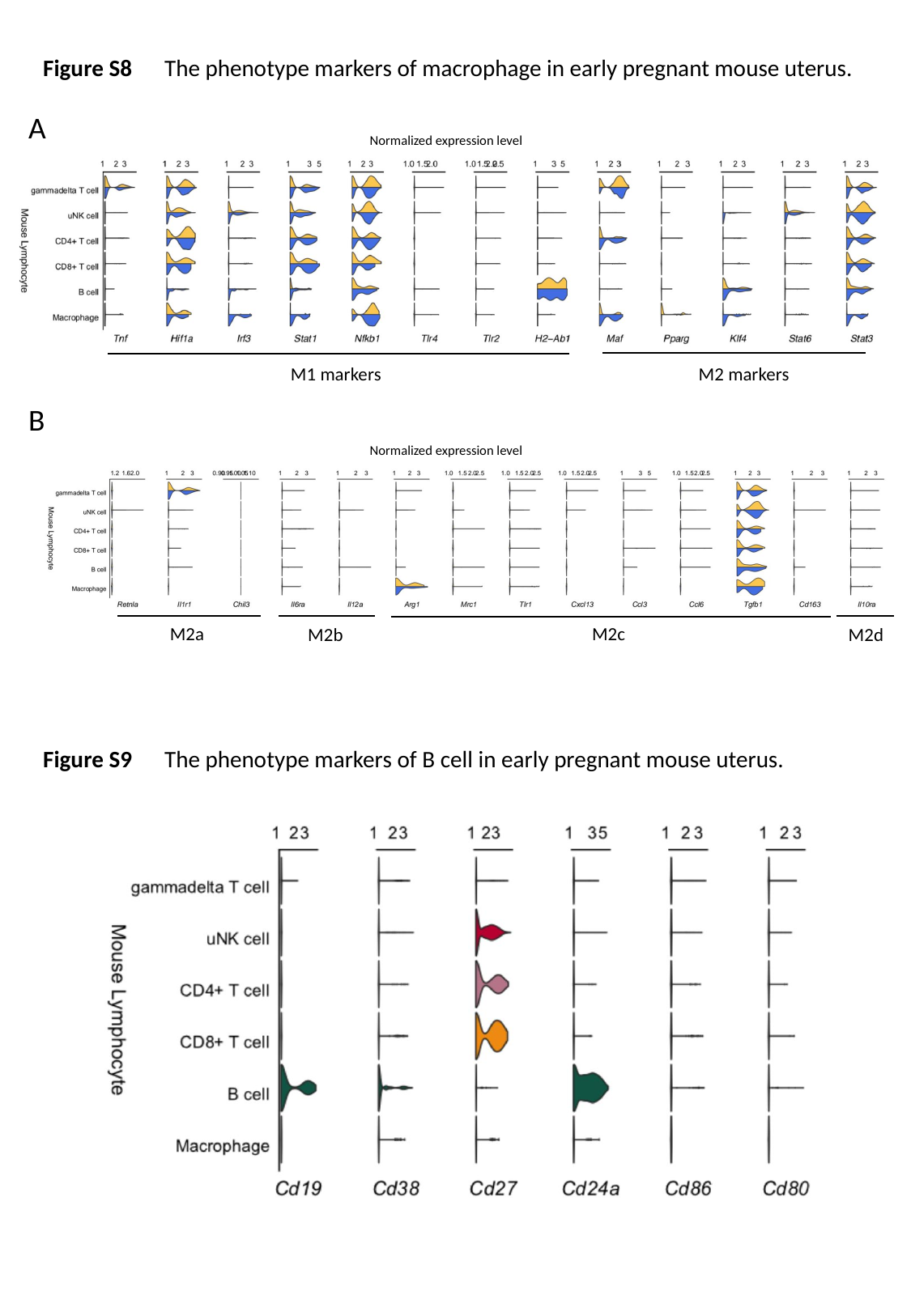

Figure S8	The phenotype markers of macrophage in early pregnant mouse uterus.
A
Normalized expression level
M1 markers
M2 markers
B
Normalized expression level
M2a
M2c
M2b
M2d
Figure S9	The phenotype markers of B cell in early pregnant mouse uterus.
