# Supplementary material for: Single-Cell RNA-Sequencing Reveals Interactions between Endometrial Stromal Cells, Epithelial Cells, and Lymphocytes during Mouse Embryo Implantation
Source: Int J Mol Sci. 2022 Dec 22;24(1):213. doi: 10.3390/ijms24010213 (PMC9820401; doi:10.3390/ijms24010213)
Supplement: Supplementary file 1 [file ijms-24-00213-s001.zip › Suppl Tables.pdf]

Table S1 The cell number of each cell type in four RNA-seq samples after passing quality control.

Cell type	IS 4.5dpc	IS 5.5dpc	I-IS 4.5 dpc	I-IS 5.5 dpc	Total
Stromal cell	2193	3801	1702	1968	9664
Epithelial cell	383	30	83	256	752
Lymphocyte	644	1049	1394	499	3586
Endothelial cell	702	641	189	355	1887
Myeloid	181	299	105	105	715
Pericyte	522	43	54	54	898
Myocyte	124	299	150	150	462
Mesothelial cell	35	67	68	68	185

Table S2 List of protein markers on different immune cell types.

Cell type	Gene marker	Reference
T cell	Cd3d, and Cd3g	(38)
Cd4+ T cell	Cd4	(38)
CD8+ T cell	Cd8	(38)
uNK cell	Klrb1c, Eomes, Itga1, and Itga2	(56, 57, 58)
B cell	Cd79a	(63)
Macrophage	Arg1, Mrc1, and Tlr1	(53)
